# Rates and predictors of switching to tenofovir alafenamide-containing ART in a nationwide cohort

**DOI:** 10.1186/s12879-019-4454-9

**Published:** 2019-10-10

**Authors:** Bernard Surial, Matthias Cavassini, Alexandra Calmy, Jan Fehr, Marcel Stöckle, Enos Bernasconi, Bianca Roth, Christoph A. Fux, Helen Kovari, Hansjakob Furrer, Andri Rauch, Gilles Wandeler, the Swiss HIV Cohort Study, A. Anagnostopoulos, M. Battegay, E. Bernasconi, J. Böni, D. L. Braun, H. C. Bucher, A. Calmy, M. Cavassini, A. Ciuffi, G. Dollenmaier, M. Egger, L. Elzi, J. Fehr, J. Fellay, H. Furrer, C. A. Fux, H. F. Günthard, D. Haerry, B. Hasse, H. H. Hirsch, M. Hoffmann, I. Hösli, M. Huber, C. R. Kahlert, L. Kaiser, O. Keiser, T. Klimkait, R. D. Kouyos, H. Kovari, B. Ledergerber, G. Martinetti, B. Martinez de Tejada, C. Marzolini, K. J. Metzner, N. Müller, D. Nicca, P. Paioni, G. Pantaleo, M. Perreau, A. Rauch, C. Rudin, A. U. Scherrer, P. Schmid, R. Speck, M. Stöckle, P. Tarr, A. Trkola, P. Vernazza, G. Wandeler, R. Weber, S. Yerly

**Affiliations:** 1 Department of Infectious Diseases, Bern University Hospital, University of Bern, Bern, Switzerland; 2 Division of Infectious Diseases, University Hospital of Lausanne, University of Lausanne, Lausanne, Switzerland; 3 Division of Infectious Diseases, Geneva University Hospital, University of Geneva, Geneva, Switzerland; 4 Division of Infectious Diseases and Hospital Epidemiology, University Hospital Zurich, University of Zurich, Zurich, Switzerland; 5 Department of Public Health, Epidemiology, Biostatistics and Prevention Institute, University of Zurich, Zurich, Switzerland; 6 Division of Infectious Diseases and Hospital Epidemiology, University Hospital Basel, University of Basel, Basel, Switzerland; 7 Division of Infectious Diseases, Regional Hospital of Lugano, Lugano, Switzerland; 8 Division of Infectious Diseases, Cantonal Hospital of St Gallen, St Gallen, Switzerland; 9 Division of Infectious Diseases, Cantonal Hospital of Aarau, Aarau, Switzerland; 10 Institute of Social and Preventive Medicine, University of Bern, Bern, Switzerland

**Keywords:** Tenofovir alafenamide, Tenofovir disoproxil fumarate, Antiretroviral therapy, Switch, Toxicity

## Abstract

**Background:**

Tenofovir alafenamide (TAF)-containing combinations were introduced in Switzerland after October 2016 and are recommended over tenofovir disoproxil fumarate (TDF) in patients with osteoporosis or impaired renal function.

**Methods:**

We included all participants of the Swiss HIV Cohort Study on TDF-containing antiretroviral therapy with follow-up visits after January 2016. We determined the proportion of switches from TDF to TAF overall, and among patients with risk factors for TDF toxicity, including osteoporosis, impaired renal function or marked proteinuria. We used multivariable logistic regression to explore predictors of switching from TDF to TAF.

**Results:**

We included 5′012 patients, of whom 652 (13.0%) had risk factors for TDF toxicity. A switch from TDF to TAF was undertaken in 2′796 (55.8%) individuals overall, and in 465 (71.3%) with risk factors. Predictors of switching to TAF were male sex (adjusted odds ratio 1.27, 95% confidence interval 1.07–1.50), age > 50 years (1.43, 1.23–1.66) and the presence of risk factors for TDF toxicity (2.21, 1.77–2.75). In contrast, patients with a non-nucleoside reverse transcriptase inhibitor (NNRTI)-based single-pill regimen (0.11, 0.09–0.13), those treated in non-tertiary care centers (0.56, 0.46–0.70), as well as those with CD4 cell counts below 500/μL (0.77, 0.66–0.90) and with chronic hepatitis C infection (0.66, 0.54–0.80) were most likely to stay on TDF.

**Conclusions:**

Over 50% of patients on TDF-containing therapy, including the majority of patients at risk for TDF toxicity, were switched to TAF within two years of its introduction in Switzerland. Individuals on NNRTI-based single-pill regimens were most likely to remain on TDF.

## Background

As AIDS-related mortality is decreasing worldwide, improving the quality of life and preventing long-term side effects of antiretroviral agents have become priorities in the management of HIV infection. When treated with antiretroviral therapy (ART), the average life expectancy of HIV-infected individuals is similar to that of uninfected individuals [1, 2]. However, life-long ART exposes persons living with HIV to treatment-associated toxicity and potential drug-drug interactions (DDI) with other medications, especially in an ageing population experiencing an increasing number of non-communicable co-morbidities [3]. Therefore, the modification of ART to regimens with minimal long-term toxicity and DDI is generally encouraged.

Tenofovir disoproxil fumarate (TDF) is a highly potent drug included in many first-line ART regimens recommended in the past decade [4]. However, its use is associated with bone mineral disorders and proximal renal tubulopathy, which can lead to impaired renal function [5–8]. Tenofovir alafenamide (TAF), a newer tenofovir prodrug, reaches high intracellular levels while exposing patients to low plasma levels, an important driver of tenofovir-related toxicity. TAF has the same antiviral activity but a better renal and bone safety profile than TDF: In registration trials, patients who switched from TDF to TAF experienced an increase in bone mineral density and a decrease in proteinuria and renal tubular markers [9, 10]. Despite the lack of long-term safety and efficacy data on the use of TAF outside of randomized controlled trials, the International Antiviral Society–USA (IAS-USA) advises against the use of TDF in patients at risk of kidney or bone disease [11]. In addition, the European AIDS Clinical Society (EACS) defines the following risk groups for which TAF should be preferred to TDF in order to prevent long-term toxicity: (a) established or high risk of chronic kidney disease; (b) concomitant use of nephrotoxic drugs, and (c) osteoporosis/osteopenia, related risk factors or a history of fragility fractures [12].

In Switzerland, TAF became available in combination with emtricitabine/elvitegravir/cobicistat (F/TAF/EVG/c, Genvoya®) in October 2016, with emtricitabine alone (F/TAF, Descovy®) in May 2017, and with emtricitabine/rilpivirine (F/TAF/RPV, Odefsey®) in July 2018. These drugs were introduced at a slightly lower price than their TDF-containing counterparts, and no generic version of TDF-containing compounds other than TDF as a single substance are available to date in Switzerland. We used data from the nationwide Swiss HIV Cohort Study (SHCS) to determine the proportion of patients with and without risk factors for TDF-related toxicity who had been switched from TDF to TAF and explored individual predictors for being switched.

## Methods

### Swiss HIV cohort study

The SHCS (www.shcs.ch) is a prospective cohort that enrolls close to 80% of all HIV-infected adults living in Switzerland [13]. Demographic, HIV-specific (e.g. date of diagnosis, most probable mode of transmission) and laboratory data (e.g. plasma viral load, CD4 cell counts, and lipid levels) are recorded at enrollment, and every 6 months thereafter. Clinical events (e.g. myocardial infarction, fragility fractures) are regularly reported using dedicated forms, and a detailed history of ART and co-medications is available for all participants. Local ethical committees of all cohort centers approved this cohort study and all patients provided written informed consent.

### Study population, definitions and outcomes

We included all HIV-infected adults with one or more clinical follow-up visits after January 1st 2016 and who were on a TDF-containing regimen for more than 30 days. Patients were classified as having switched if TAF was introduced within 90 days after stopping TDF, and if TAF was continued for at least 14 days. To restrict our analysis to direct switches from TDF to TAF, we excluded patients who were prescribed abacavir during the time between the use of TDF and TAF. The decision to switch or not was at the discretion of the treating physician, and no additional guidance was given. Database closure was on the 1st of August 2018.

Our primary aim was to describe the proportion of patients having switched from TDF to TAF and to identify related predictors. Additionally, the same outcomes were explored in patients “at risk of TDF toxicity”, defined as the presence of at least one of the following risk factors: [1] estimated glomerular filtration rate (eGFR) < 60 mL/min, [2] marked proteinuria (protein-to-creatinine ratio ≥ 50 mg/mmol [≥500 mg/g]) or [3] osteoporosis (T-score ≤ − 2.5 in any bone mineral density measurement and/or occurrence of fragility fractures). Our secondary aims were to explore the reasons for discontinuing TDF, as reported by treating physicians, using pre-defined stopping reasons which are systematically recorded through an online tool. Finally, we determined whether TAF was used together with drugs that are categorized as “do not co-administer” for TAF but not TDF according to the Liverpool Drug Interaction Group Database [14], in order to evaluate potential problematic DDI with TAF.

Arterial hypertension was defined as at least two measurements > 140 mmHg systolic or > 90 mmHg diastolic and/or currently being on antihypertensive treatment, diabetes mellitus as HbA1c ≥6.5% and/or currently being on antidiabetic treatment and dyslipidemia as total-cholesterol to HDL ratio > 5 and/or currently being on lipid lowering treatment. Chronic hepatitis B virus (HBV) infection was defined by the presence of a positive hepatitis B surface antigen, and hepatitis C infection (HCV) as a detectable HCV viral load at any time-point, irrespective of HCV treatment. A history of cardiovascular disease included the previous occurrence of any of the following: myocardial infarction, ischemic stroke, arterial interventions including coronary angioplasty/stenting, coronary artery by-pass grafting and venous thromboembolism.

### Statistical analyses

We compared baseline demographic and clinical characteristics, comorbidities and laboratory values between patients “at risk of TDF toxicity” and those without risk factors using Chi-square, Wilcoxon rank sum and t-tests, where appropriate. Baseline was defined as [1] switching date for patients on TAF, [2] October 1st 2016 for patients remaining on TDF, or [3] registration date for patients remaining on TDF who joined the SHCS after October 1st 2016. Multivariable logistic regression was used to explore risk factors of switching to TAF, and included the following explanatory variables: sex, age (< 50 or ≥ 50 years), region of origin, education status, CD4 cell count (< 500 or ≥ 500/μL), follow-up in a tertiary versus non-tertiary center (including private physicians), comorbidities (chronic HBV or HCV infections, history of cardiovascular disease, diabetes, arterial hypertension and dyslipidemia), the presence of at least one risk factor for TDF associated toxicity, and use of boosted protease inhibitors (PI) or non-nucleoside reverse transcriptase inhibitor (NNRTI)-based single-pill regimens. Osteoporosis was excluded from the multivariable analyses because bone mineral density measurements were only available in one third of patients. However, we repeated the main analyses in patients with at least one risk factor for TDF toxicity, including osteoporosis and eGFR. Odds ratios from multivariable analyses are presented in Forest plots, stratified by the presence or absence of any risk factors for TDF toxicity. All statistical analyses were performed using Stata 15.1 (Stata Corp, College Station, TX, USA).

## Results

### Study population

Of 10′246 HIV-infected individuals in active follow-up after January 1st 2016, 8′245 (80.5%) ever received TDF for more than 30 days. After excluding patients who switched to abacavir or initiated TAF more than 90 days after stopping TDF (*n* = 3′233), 5′012 patients remained in our study population. Overall, 3′645 (72.7%) participants were male, 789 (15.7%) were of African origin, and the median age was 49 years (interquartile range [IQR] 41–56). Measurements of creatinine and proteinuria at baseline were available for 4′813 (96.0%) and 3′102 (61.9%) patients respectively, and a bone densitometry measurement was available in 1′624 (32.4%) individuals. In total, 652 patients (13.0%) had one or more risk factors for TDF-associated toxicity: 243 had an eGFR < 60 mL/min (4.8%), 154 (3.1%) had marked proteinuria, and osteoporosis was diagnosed in 325 (6.5%) patients. Table 1 summarizes the main demographic and clinical characteristics of the study population, stratified by the presence of at least one risk factor for developing TDF-related toxicity. Patients with risk factors were older, less likely to be of African origin, but more likely to be persons who inject drugs (PWID) and to have arterial hypertension, dyslipidemia, diabetes or a history of cardiovascular disease.

**Table 1:** Characteristics of the study population at baseline^a^

Characteristics	No TDF-toxicity risk N = 4′360	TDF-toxicity risk *N* = 652	*P*
Male sex (%)	3194 (73.3)	451 (69.2)	0.03
Median age in years (IQR)	48 (40–54)	56 (50–62)	< 0.001
African origin (%)	742 (17.0)	47 (7.2)	< 0.001
High-level education (%)	1471 (33.7)	181 (27.8)	0.01
Transmission group (%)			< 0.001
MSM	2071 (48.7)	270 (42.4)	
PWID	396 (9.3)	108 (17.0)	
other	1789 (42.0)	259 (40.7)	
Median CD4 count in cells/μL (IQR)	640 (486–828)	615 (443–826)	0.01
Median CD4 nadir in cells/μL (IQR)	226 (122–334)	171 (75–273)	< 0.001
Type of center (%)			0.44
Tertiary care center	2256 (51.7)	348 (53.4)	
Other	2104 (48.3)	304 (46.6)	
Chronic HBV infection (%)	291 (7.1)	47 (7.5)	0.71
Chronic HCV infection (%)	509 (11.9)	115 (18.0)	< 0.001
History of CV disease (%)	293 (6.7)	95 (14.6)	< 0.001
Diabetes (%)	245 (5.6)	77 (11.8)	< 0.001
Arterial hypertension (%)	2264 (51.9)	405 (62.1)	< 0.001
Dyslipidemia (%)	1′864 (42.8)	345 (52.9)	< 0.001
Median eGFR in mL/min (IQR)	93.8 (80.6–106.3)	73.0 (56.3–95.5)	< 0.001
eGFR category (%)			< 0.001
> 90 mL/min	2390 (57.3)	198 (30.9)	
60–90 mL/min	1782 (42.7)	200 (31.2)	
< 60 mL/min	0	243 (37.9)	
Proteinuria (%) [*n* = 3102]			< 0.001
< 15 mg/mmol	1920 (72.5)	139 (30.6)	
15–50 mg/mmol	727 (27.5)	162 (35.6)	
> 50 mg/mmol	0	154 (33.9)	
Osteoporosis (%)			n.a.
T-score ≤ −2.5 [*n* = 1624]	0	273 (41.9)	
Fragility fracture	0	77 (11.8)	
Combined	0	325 (49.9)	
PI-based ART (%)	994 (22.8)	184 (28.2)	0.01
NNRTI-based single-pill regimen (%)	1755 (40.3)	223 (34.2)	0.01

*TDF* Tenofovir disoproxil fumarate, *IQR* Interquartile range, *MSM* Men having sex with men, *PWID* Persons who inject drugs, *HBV* Hepatitis B virus, *HCV* Hepatitis C virus, *CV* Cardiovascular, *eGFR* Estimated glomerular filtration rate, *PI* Protease inhibitor, *ART* Antiretroviral treatment, *NNRTI* Non-nucleoside reverse transcriptase inhibitor

^a^time of switch for those who switched, or 1st October 2016 (introduction of TAF in Switzerland) or registration date if registered after that date for those who did not switch

TDF toxicity risk defined as presence of a least one of the following risk factors: eGFR < 60 mL/min, urine protein-to-creatinine ratio of ≥50 mg/mmol or osteoporosis

### Rates and predictors of switching from TDF to TAF

Nearly two years after the introduction of TAF in Switzerland, 2′796 (55.8%) individuals had TDF replaced by TAF, with large differences across cohort centers, ranging from 32.6 to 65.3% (*p* < 0.001). Overall, most individuals received F/TAF and a third drug (*n* = 1′635, 58.5%), followed by F/TAF/EVG/c (n = 1′116, 39.9%) and F/TAF/RPV (*n* = 45, 1.6%). In those who received F/TAF, the most common third drug was dolutegravir (661, 40.4%), followed by boosted darunavir (277, 16.9%), nevirapine (243, 14.9%) and raltegravir (130, 8%). In 1475 (52.8%) instances, the switch from TDF to TAF was the only modification in the ART regimen with all other components remaining unchanged.

Of the 652 patients with at least one risk factor for TDF-associated toxicity, 465 (71.3%) were switched to TAF. Patients switched at a median of 9.7 months (IQR 7.1–12.2 months) after the introduction of TAF into the Swiss market, without a significant difference between those with or without TDF-toxicity risk factors (9.5 versus. 9.7 months, *p* = 0.15). Thirty-four patients had access to TAF prior to its official licensing, either through an early access program or within a study protocol, and remained in our study population.

**Fig. 1:**
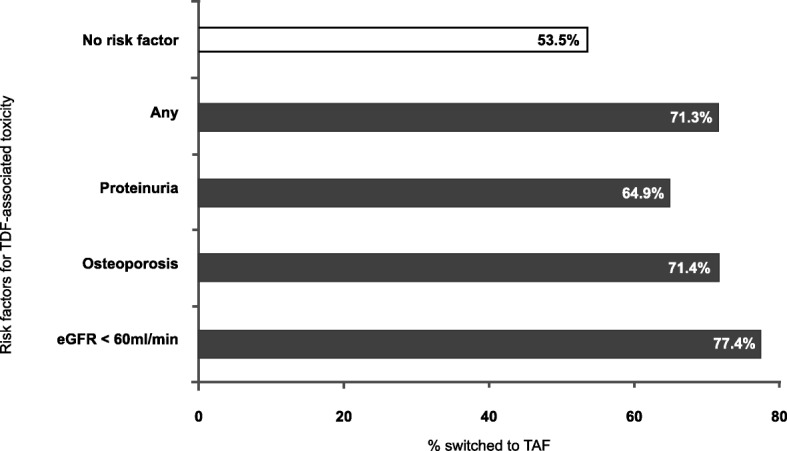
Rates of switching from TDF to TAF, according to risk factor. *eGFR* Estimated glomerular filtration rate, *TDF* Tenofovir disoproxil fumarate, *TAF* Tenofovir alafenamide. Proteinuria defined as urine protein-to-creatinine ratio > 50 mg/mmol

In multivariable analyses, patients were more likely to switch to TAF if they were male, older than 50 years, had at least one risk factor for TDF toxicity, or arterial hypertension. In contrast, patients with CD4 cell counts below 500/μL, those followed in non-tertiary centers, and HCV-coinfected individuals were more likely to remain on TDF. An important predictor for staying on TDF was the use of NNRTI-based single-pill regimens (Table 2), with comparable estimates for efavirenz and rilpivirine based single-tablet regimens. Compared to the population without risk factors, the proportion of individuals who switched to TAF was higher in the presence of an eGFR < 60 mL/min (77.4%), osteoporosis (71.4%), or marked proteinuria (64.9%, Fig. 1). In patients with an eGFR < 60 mL/min who remained on TDF, median eGFR at baseline was 56.5 mL/min (IQR 52.6–58.0 mL/min) and none had an eGFR ≤30 mL/min.

**Table 2:** Predictors of switching from TDF to TAF

			Multivariable Analysis	
Variable	*N* (%)	Switched (%)	Adjusted odds ratio (95% CI)	*P*
Male sex	3′645 (72.7)	2′102 (57.7)	1.27 (1.07–1.50)	0.01
Age > 50 years	2′235 (44.6)	1′413 (63.2)	1.43 (1.23–1.66)	< 0.001
African origin	789 (15.7)	377 (47.8)	0.83 (0.67–1.01)	0.07
High level education	1′652 (33.0)	923 (55.9)	1.16 (1.00–1.35)	0.05
CD4 < 500/μL at baseline^a^	1′406 (28.1)	754 (53.6)	0.77 (0.66–0.90)	< 0.001
Follow-up in a non-tertiary care center	2′408 (48.0)	1′169 (48.6)	0.56 (0.46–0.70)	< 0.001
Comorbidities				
Chronic HBV infection	338 (6.7)	188 (55.6)	0.97 (0.74–1.26)	0.81
Chronic HCV infection	624 (12.5)	349 (55.9)	0.66 (0.54–0.80)	< 0.001
Diabetes	322 (6.4)	202 (62.7)	0.95 (0.71–1.26)	0.70
Arterial hypertension	2′669 (53.3)	1′584 (59.4)	1.21 (1.04–1.40)	0.01
History of CV disease	388 (7.7)	243 (62.6)	0.79 (0.61–1.03)	0.08
Dyslipidemia	2′209 (44.1)	1′336 (60.5)	1.09 (0.94–1.26)	0.26
At least one risk factor for TDF toxicity^b^	652 (13.0)	465 (71.3)	2.21 (1.77–2.75)	< 0.001
PI-based ART	1′178 (23.5)	855 (72.6)	0.97 (0.81–1.16)	0.74
NNRTI-based single-pill regimen	1′978 (39.5)	522 (26.4)	0.11 (0.09–0.13)	< 0.001

*TDF* Tenofovir disoproxil fumarate, *TAF* Tenofovir alafenamide, *HBV* Hepatitis B virus, *HCV* Hepatitis C virus, *CV* Cardiovascular, *PI* Protease inhibitor, *ART* Antiretroviral treatment, *NNRTI* Non-nucleoside reverse transcriptase inhibitor

^a^time of switch for those who switched, or 1st October 2016 (introduction of TAF in Switzerland) or registration date if registered after that date for those who did not switch

^b^osteoporosis, impaired renal function or marked proteinuria

**Fig. 2:**
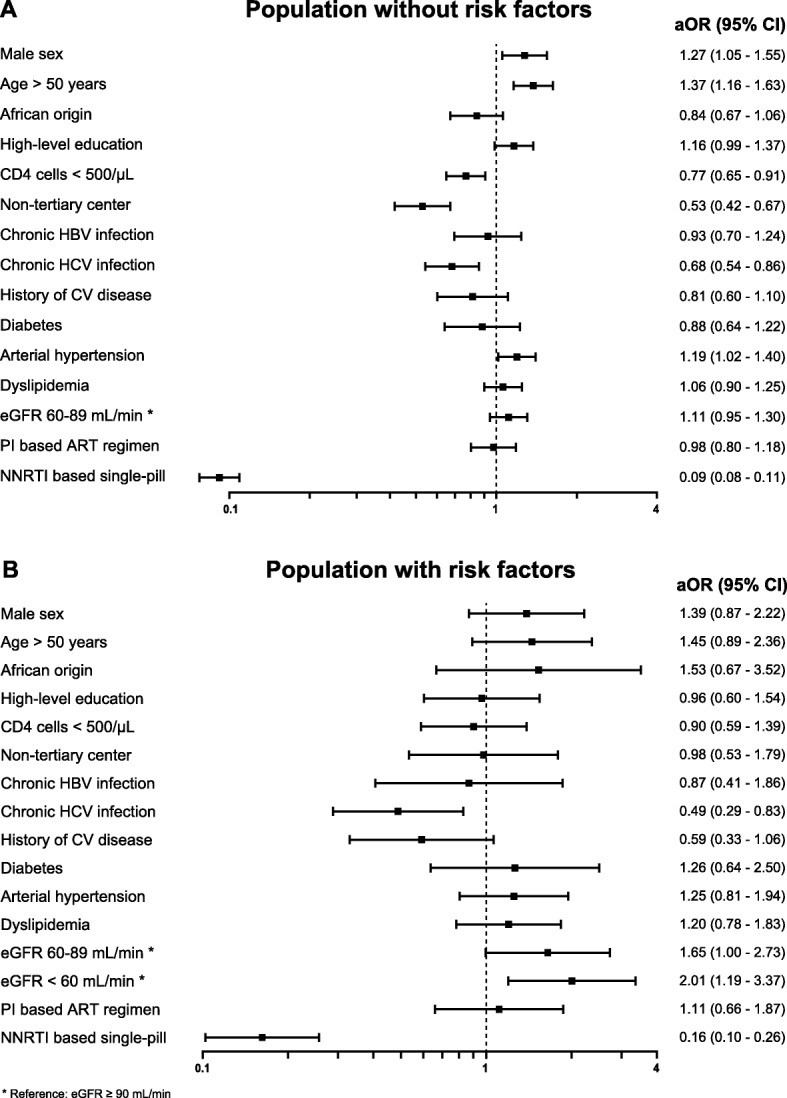
Probability of switching from TDF to TAF. Adjusted odds ratios (aOR) and 95% confidence intervals (CI) in study population without (Panel **a**) and with risk factors for TDF-associated toxicity (Panel **b**). *aOR* Adjusted odds ratio, *HBV* Hepatitis B virus, *HCV* Hepatitis C virus, *CV* Cardiovascular disease, *eGFR* Estimated glomerular filtration rate, *PI* Protease inhibitor, *ART* Antiretroviral treatment, *NNRTI* Non-nucleoside reverse transcriptase inhibitor

In analyses restricted to patients with TDF-related risk factors, the associations between switching to TAF and renal impairment, the use of NNRTI-based single-pill regimens and co-infection with HCV remained statistically significant. In contrast, higher age, male sex or the presence of arterial hypertension were not associated with switching to TAF among persons with TDF-related risk factors. (Fig. 2a and b). The most common ART regimens in patients staying on TDF despite TDF-related risk factors were fixed-dose combinations of F/TDF/RPV (31.6%) and F/TDF/efavirenz (25.1%).

### Reasons for switching from TDF to TAF and presence of drug-drug interactions

The main reasons for replacing TDF by TAF, as reported by SHCS physicians, were “prevention of expected side-effects” (24.9%), followed by “simplification of current treatment” (9.2%), and “presence of established kidney toxicity” (8.2%) (Table 3). Less specific reasons such as patient’s wish and physician’s decision were given in 1′372 cases (49.1%). The use of drugs which are contraindicated with TAF was rare in our cohort: Three individuals switched to TAF (all once-daily) while taking rifabutin, and one patient switched while taking carbamazepine. Within the group of patients that remained on TDF, treatment with rifampicin (*n* = 12), rifabutin (*n* = 14) and carbamazepine (*n* = 3) were more common. No patient in the study population received phenytoin.

**Table 3:** Reasons for switching from TDF to TAF

TDF stopping reason	No TDF-toxicity risk (*n* = 2′331)	TDF-toxicity risk (*n* = 465)	Total (*n* = 2′796)
Prevention of expected side-effects	614 (26.3%)	83 (17.9%)	697 (24.9%)
Established kidney toxicity	147 (6.3%)	83 (17.9%)	230 (8.2%)
Toxicity, other	124 (5.3%)	34 (7.3%)	158 (5.7%)
Treatment simplification	223 (9.6%)	34 (7.3%)	257 (9.2%)
Drug-drug interaction	15 (0.6%)	3 (0.7%)	18 (0.6%)
Patient’s wish	195 (8.4%)	48 (10.3%)	243 (8.7%)
Physician’s decision	375 (16.1%)	88 (18.9%)	463 (16.6%)
Other reason, not specified	581 (24.9%)	85 (18.3%)	666 (23.8%)
Missing	57 (2.5%)	7 (1.5%)	64 (2.3%)

*TDF* Tenofovir disoproxil fumarate, *TAF* Tenofovir alafenamide.

TDF toxicity risk defined as presence of a least one of the following risk factors: eGFR < 60 mL/min, urine protein-to-creatinine ratio of ≥50 mg/mmol or osteoporosis

## Discussion

Two years after the introduction of TAF in Switzerland, 56% of all patients on TDF-containing ART overall, and 72% of those with risk factors for TDF-associated toxicity had TDF replaced by TAF. The highest proportions of patients switched to TAF were found in men, those older than 50 years, as well as in individuals with renal impairment, whereas the use of a NNRTI-based single-pill regimen was predictive of staying on TDF. The main reason for switching to TAF, as reported by treating physicians, was the prevention of potential side effects due to TDF. Our study underlines the importance of monitoring treatment changes and related predictors in order to evaluate a program’s potential to react to changes in guidelines and new drug availability, as well as to foster patient-centered management.

Although the majority of individuals on TDF-containing ART were switched to TAF, we found large differences in the use of TAF across treatment sites and between tertiary and non-tertiary sites. Similar diversity in patterns of treatment changes were observed across other studies: in a cohort of 444 patients on TDF-containing ART in Germany, only 34% of all patients switched to TAF-containing ART within 2 years of its introduction [15]. In contrast, a retrospective analysis of four treatment centers in the US showed that of all switches performed within the first year after TAF became available, 86% received TAF as the new drug [16]. The lower cost of F/TAF compared to F/TDF in countries without generic F/TDF could partly explain these results: for instance, generic F/TDF was available in Germany but not in the US and Switzerland. Furthermore, some physicians may have decided to wait for the availability of generic F/TDF instead of switching individuals without risk factors for TDF-toxicity to TAF. Eventually, these generic compounds never made it to the market in Switzerland due to legal issues.

Our study highlights the multitude of factors which potentially play a role in shaping physicians’ choices and patient preferences in terms of ART modalities. Although only 8.2% of all switches in our study could be attributed to the presence of established kidney dysfunction, as reported by treating physicians, a low eGFR (especially below 60 mL/min) was a predictor for receiving TAF. This finding is in line with previous reports [16–18] and such a strategy is supported by the results of randomized controlled trials in patients with renal impairment [10, 19]. The presence of boosted PI-based regimens was not associated with an increase in switching to TAF in our study, even though these patients are at increased risk of TDF-associated toxicity. In a randomized controlled trial, Goicoechea et al. showed a faster decline in renal function when TDF was co-administered with boosted PI, possibly due to increased tenofovir plasma levels [20]. Similarly, a meta-analysis of randomized trials only favored TAF over TDF for renal outcomes when a booster was co-administered [21]. The single most important predictor of staying on TDF was the use of NNRTI-based single-pill regimens. For these regimens, TAF-containing counterparts were either unavailable as a single-pill or introduced late during the observation period. We hypothesize that even in the presence of risk factors, patients and physicians may have preferred to keep the convenient and well-tolerated single-pill regimen in order to secure adherence and virological efficacy. Many patients without risk factors for TDF-toxicity remained on TDF in our cohort, a practice supported by the EACS guidelines [12] and current data which are insufficient to recommend TAF over TDF for these patients. Since TDF is available in generic form in many countries, its use could considerably reduce HIV-related costs.

In general, the potential for DDI of TDF and TAF is similar. However, drugs such as carbamazepine, phenytoin, rifampicin or rifabutin seem to be more problematic when combined with TAF than with TDF [14]. For instance, the administration of TAF in combination with rifampicin led to significantly lower intracellular and plasma levels of tenofovir in pharmacokinetic studies [19, 22]. While twice-daily dosing of TAF is sufficient to overcome the decrease in plasma levels induced by rifampicin and rifabutin, carbamazepine or phenytoin seem to decrease tenofovir plasma levels to an extent that TAF can lose its therapeutic effect. Despite the nearly systematic switch to TAF in some of our study centers, only one of 2′796 patients who switched to TAF was receiving a contra-indicated co-medication (carbamazepine). Considering the very low number of inadequate drug combinations after the switch to TAF, our study shows that the event of significant DDI in the context of single-drug substitutions remains very low when HIV physicians are familiar with important DDI mechanisms.

The large number of patients analyzed during the months following the registration of TAF in Switzerland allowed us to explore the main drivers of ART changes in response to the availability of drugs with an improved toxicity profile. Furthermore, the presence of detailed information on treatment changes and stopping reasons helped us improve our understanding of the determinants of switching from TDF- to TAF-containing ART. However, although this study is representative for current treatment strategies in Switzerland, extrapolations to other health care systems should be undertaken with caution. Additionally, given the large number of potentially nephrotoxic drugs with complex toxicity profiles taken by our patients, we could not include comprehensive information on their use in our analysis. As the prescription of such drugs may have motivated physicians to recommend switching to a TAF-containing regimen, we may have failed to grasp the reason for switching in some cases. Similarly, as the assessment of bone mineral density was available in only 32% of our study population, we may have underestimated the proportion of patients at risk of TDF-toxicity. The potential under-estimation of osteoporosis in our cohort was mitigated by our inclusion of data on fragility fractures systematically collected within the SHCS. Since TAF is not recommended during pregnancy, the desire to have children may have influenced treatment choices in some women and led to lower switching rates among this group of patients. Finally, information about physicians’ motivation to recommend a treatment change to their patients was limited to predefined stopping reasons and were inconclusive in almost 50% of cases. Such information would have been important to better understand the large differences in TAF prescriptions between tertiary centers and other health care facilities.

## Conclusions

The majority of patients at risk for TDF-toxicity were switched from TDF to TAF within two years after its introduction in Switzerland. Among 30% of patients at risk of TDF-toxicity who did not switch during this time-period, being on a NNRTI-based single-pill regimen seemed to be an important reason for remaining on TDF. However, large differences across clinics were observed and related reasons should be further explored. Since current recommendations on switching to TAF are based on limited data from selected groups of patients in randomized controlled trials, further data on clinical outcomes after the introduction of TAF in cohorts such as ours remain essential to inform optimal patient management.

## Data Availability

The datasets used and/or analyzed during the current study are available from the corresponding author on reasonable request.

## Abbreviations

ART: Antiretroviral therapy
DDI: Drug-drug interaction
EACS: European AIDS Clinical Society
eGFR: Estimated glomerular filtration rate
HBV: Hepatitis B virus
HCV: Hepatitis C virus
IAS-USA: International Antiviral Society-USA
IQR: Inter-quartile range
NNRTI: Non-nucleoside reverse transcriptase inhibitor
PI: Protease inhibitors
PWID: Persons who inject drugs
SHCS: Swiss HIV Cohort Study
TAF: Tenofovir alafenamide
TDF: Tenofovir disoproxil fumarate
